# Health service barriers to HIV testing and counseling among pregnant women attending Antenatal Clinic; a cross-sectional study

**DOI:** 10.1186/1472-6963-14-267

**Published:** 2014-06-19

**Authors:** Golda Dokuaa Kwapong, Daniel Boateng, Peter Agyei-Baffour, Ernestina A Addy

**Affiliations:** 1 The United States Agency for International Development (USAID)/Focus Region Health Projects, Accra, Ghana; 2 Department of community Health, Kwame Nkrumah University of Science and Technology, Kumasi, Ghana

**Keywords:** HTC, PMTCT, HIV, Pregnant Women, Ghana

## Abstract

**Background:**

HIV testing and counseling (HTC) remains critical in the global efforts to reach a goal of universal access to prevention and timely human immunodeficiency virus (HIV) treatment and health care. Routine HIV testing has been shown to be cost-effective and life-saving by prolonging the life expectancy of HIV patients and reducing the annual HIV transmission rate. However, these benefits of routine HIV testing may not be seen among pregnant women attending antenatal clinic (ANC) due to health facility related factors. This paper presents the influence of health facility related factors on HTC to inform HTC implementation.

**Methods:**

The study was cross-sectional in design and used structured questionnaire and interview guides to gather information from 300 pregnant women aged 18 to 49 years and had attended ANC for more than twice at the time of the study. Twelve health workers were interviewed as key informants. Respondents were selected from the five sub metro health facilities in the Kumasi Metropolis through systematic random sampling from August to November 2011. Pregnant women who had not tested after two or more ANC visits were classified as not utilizing HTC. Data was analyzed with STATA 11. Logistic regression was run to assess the odds ratios at 95% confidence level.

**Results:**

Twenty-four percent of the pregnant women had not undergone HTC, with “never been told” emerging as the most cited reason as reported by 29.5% of respondents. Decisions by pregnant women to take up HTC were mostly influenced by factors such as lack of information, perceptions of privacy and confidentiality, waiting time, poor relationship with health staff and fear of being positive.

**Conclusions:**

Access to HTC health facility alone does not translate into utilization of HTC service. Improving health facility related factors such as health education and information, confidentiality, health staff turnaround time and health staff-client relationship related to HTC will improve implementation.

## Background

The World Health Organization (WHO) estimated 33.3 million people living with HIV/AIDS in 2009 with 2.3 million coming from sub Saharan Africa [1]. The effects of Human Immunodeficiency virus (HIV) and acquired immune deficiency syndrome (AIDS) on individuals and families have been devastating, with tragedies, untimely deaths, medical, financial and social burdens [2]. Efforts such as prevention of mother-to-child transmission (PMTCT) and the use of antiretroviral are underway globally, to minimize the burden. However, HIV/AIDS is still a growing problem in many parts of the world especially Sub-Saharan Africa with women, foetus and children being most vulnerable [3].

It is worthy to note that a majority of adults living with HIV (61%) are women and thus mother-to-child transmission of HIV is eminent. Mother-to-child transmission (MTCT) of HIV/AIDS is almost eliminated in high-income countries with the help of safe delivery and breastfeeding practices, access to antiretroviral therapy and effective HIV Testing and Counseling (HTC) [4]. With these interventions fully implemented globally, about a thousand lives of children could be saved annually [3]. Effective PMTCT requires a three-fold strategy; (1) preventing HIV infection among prospective parents by making HIV testing and other prevention interventions available in the populace (2) avoiding unwanted pregnancies among HIV positive women by providing appropriate counseling on contraception and (3) the use of prophylactic antiretroviral during pregnancy as well as other interventions aimed at reducing the risk of vertical transmission [5].

HTC is very important in efforts to ensure universal access to prevention and timely HIV treatment and care services. Previous studies have shown that HTC could be cost-effective and could increase the life expectancy of individuals with HIV [6] and it is a key factor in the PMTCT. It also provides clients the opportunity to confidentially learn of the HIV status, which is a gateway to accessing treatment [7,8]. Utilization of HTC has however been low especially in sub Saharan Africa and as at 2008, about 80% of adults living with HIV in sub Saharan Africa did not know of their status [9].

HTC by the Ghana National AIDS Control Programme (NACP) was established to assist those who do not know their status, infected, affected and uninfected to manage their lives well to help prevent the spread of the disease in Ghana and Africa as a whole. A few of the groups mentioned above patronize HTC whiles majority does not due to lack or inadequate of counseling skills, lack of rapport with clients and poor patient management skills. In 2009, only 40% of ANC clients were counseled and tested for HIV and only 28% of those estimated to be in need of ART for PMTCT received it [10]. In 2009, only 29% of ANC clinics in the country provided PMTCT services to its clients [11]. Although ANC attendance is high, it has not translated into the number of pregnant women who go for HTC services despite efforts to provide free HTC services. A recent study on HIV positive women’s perception of ART centres revealed that, unavailability of drugs and lack of follow ups are key barriers to utilization of PMTCT services [12]. There are limited studies however to evaluate the factors that account for the wide ANC-HTC gap. This study sought to investigate the influence of health facility related factors on HTC among pregnant women presenting at ANC in the Kumasi metropolis, Ghana.

## Methods

### Study design and setting

The study was cross sectional in design with both qualitative and quantitative methods. These methods were chosen because combining qualitative and quantitative methods elicit in-depth information and generate extensive discussions on contextual explanations on women’s perceived barriers to HTC [13]. The study was conducted in the Kumasi metropolis because it accounts for a third of population in Ashanti region (2009 projection, 1,889,934) and second urbanized after Greater Accra in Ghana. It is located almost at the centre of Ghana, an economic nerve and has varying health facilities.

The Metropolitan Health Services is decentralized and are organized around five (5) Sub Metro Health Teams; namely, Bantama, Asokwa, Manhyia North, Manhyia South and Subin. The Metro Health Team is led by its Director of Health Services who has the overall responsibility for planning, monitoring and evaluating the performance of the Health Sector in the metropolis. The city has many public and private health facilities with one teaching hospital, the Komfo Anokye Teaching Hospital (KATH), one of the three national autonomous hospitals, four (4) quasi health institutions, five (5) health centres owned by the Church of Christ and the Seventh-Day Adventist Church. In 2010 and 2011, a total of 97,852 and 122,708 ANC registrants were recorded in the Kumasi metropolis of which 79% and 88% respectively tested and received post-test counseling [14]. As at 2011, there were 285 counseling and testing (CT) and 268 PMTCT centres in the Ashanti region [14].

### Study population and sample

The study was conducted in five sub metro health facilities providing HTC services in the Metropolis. These were Kumasi South Government Hospital (62 respondents), Suntreso Government Hospital (68 respondents), Tafo Government Hospital (56 respondents), Maternal and Child Health Hospital (56 respondents) and Manhyia Government Hospital (58 respondents). Pregnant women who had attended ANC more than twice and HIV counsellors at ANC in the selected health facilities in the Kumasi Metropolis were studied.

The sample size was determined following [15] as

(1) $$n=\frac{Z^{2}\mathrm{pq}}{d^{2}}$$

Where *n* is the sample size; z is the reliability co-efficient (1.96) at 95% confidence level, d the allowable error margin (0.05), p is the proportion of women in fertility age (WIFA) in the population (23%) and q = 1-p. This gave an approximate sample size of 300.

Simple random sampling ballot in which health facilities offering PMTCT services in each sub-metro were numbered was used to select study facilities. Five facilities, one each of the five sub-metros were selected without replacement. In each of the selected health facility, the study was explained to all pregnant women presenting at the antenatal clinic after which pieces of papers with inscriptions, ‘YES’ and ‘NO”, were put in a box for pick by respondents. Respondents who picked ‘YES’ and consented to participate in the study were enrolled till the required sample size of 300 for the quantitative survey was reached.

### Data collection and analysis

#### Quantitative

The data collection technique was interviewing where trained research assistants interviewed respondents with structured questionnaires. Data was collected from August to November 2011. Questionnaires and interview results were checked for completeness and internal errors during data collection. Questionnaires were then sorted, numbered and kept in files labeled per facility from which the participants were recruited. Data was then coded before entering into SPSS. The perception on quality of PMTCT services and its influence on HIV Counseling and Testing was analysed using logistic regressions. This was done using STATA 11. The dependent variable was utilization of HTC and independent variables were facility related factors that influence HTC and these included waiting time, privacy and confidentiality, feeling attended to, being listened to, being treated with respect and trust for health workers. Pregnant women who had not been tested for HIV after two or more ANC visits were classified as not utilizing HTC and this was coded as 1.

#### Qualitative

Focus group discussions (FGDs) and in-depth interviews using interview guides and tape recorders were the data collection techniques and tools respectively. Five FGDs, involving 40 pregnant women (8 per group per FGD) were conducted. They involved three (3) groups of women who had not undertaken HTC and two (2) groups of pregnant women who had undergone HTC. Topics discussed involved various health facility barriers to HTC. Twelve health workers were also involved in the in-depth interviews. Qualitative data was analyzed using ATLAS.ti. Audio-recorded data from both FGDs and key informants interviews were transcribed verbatim and translated into English. Data was analysed thematically and salient quotes from the themes were presented as results.

### Ethical considerations

The Committee for Human Research Publication and Ethics of the School of Medical Science, Kwame Nkrumah University of Science and Technology (KNUST) gave ethical clearance. Participants were taken through consenting processes prior to enrolment into the study. There was full disclosure or discussion of relevant information and questions related to the study. Also participants who could not read were informed about the study by translating the consenting information into their local language for adequate comprehension. **T**hey were told that enrolment into the study was voluntary. No response was related to participants who were assigned study identification numbers.

## Results

### Background characteristics of respondents

The background characteristics of respondents are presented in Table 1. The mean age of respondent was 28.07 years (±5.32) years with a majority, 59.3%, falling within the ages of 25 - 34 years. A majority, 79.9% were married and 83.7% were Christians and about 60% had JSS/middle school education while 11.4% had no formal education. A little over two-thirds, 64.3%, were employed and 58.7% had a family size of between 3 and 5.

**Table 1:** Respondents’ background characteristics

**Variables**	**Frequency**	**Percentage (%)**
**Age (n = 300)**		
− < 25	82	27.3
− 25 – 34	178	59.3
− 35 – 44	38	12.7
− > 45	2	0.7
**Marital status (n = 299)**		
− Married	239	79.7
− Single	60	20.3
**Religion (n = 299)**		
− Christian	251	83.9
− Moslem	48	16.1
**Education (n = 299)**		
− No formal	34	11.4
− Primary	20	6.7
− JSS/middle	176	58.8
− Sen. Sec/Tech	61	20.4
− Tertiary	8	2.7
**Employment status (n = 300)**		
− Employed	193	64.3
− Not employed	107	35.7
**Family size (n = 300)**		
− <3	104	34.7
− 3 – 5	176	58.7
− > 5	20	6.6

### Utilization of HIV testing and counseling

**Figure 1:**
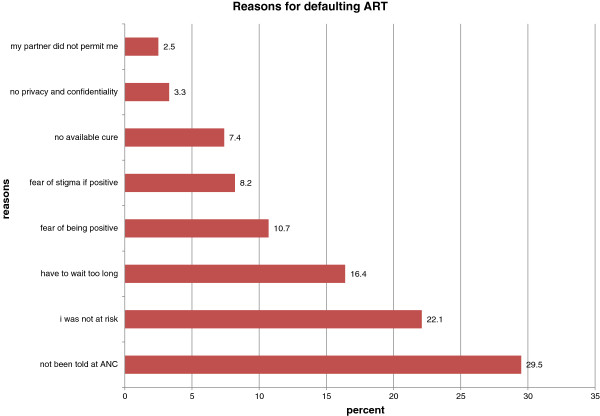
Reasons for defaulting ART.

Two hundred and twenty-seven pregnant women representing 76% of the respondents had gone through HIV testing and counseling while 73 (24%) had not.Figure 1 presents respondents’ reasons for not utilizing HTC. “I have not been told” emerged as the most cited reason why a pregnant woman had not undergone HIV testing and counseling as expressed by 29.5% of respondents. Only 2.5% had not tested because their partners had not permitted them. Other reasons cited were “I am not at risk” (22.1%), “long waiting time” (16.4%), “fear of being positive” (10.7%), “fear of stigma if positive” (8.2%), “No available cure if positive” (7.4%) and “no privacy and confidentiality at facility” (3.3%).

Although HTC was high in some of the health facilities, participants from these facilities disclosed they had to do it because they were told it was compulsory they were coerced to do it. They explained that the midwives told them that if they do not do the testing, they could not deliver in that facility as a participant reported:

*“As for this place they force us to do anything. When we came, the midwife told us ‘if you do not do HIV testing, you cannot deliver your baby here.’ And me too I like this place so I have to do it by force”* (Pregnant women, Focus group discussion, health facility in Kumasi).

Some participants disclosed they took the test without their knowledge. They said before they realized it was for HIV, their blood samples had already been taken. A respondent had this to say;

*“I didn’t even know it was HIV testing. When I came they took my card and called me in to go for test. So I asked the one who took my blood sample what it was for, before I was told it was for HIV. […] I think this is not good”* (Pregnant women, Focus group discussion, health facility in Kumasi).

Some health workers also disclosed that sometimes the use of force becomes the only alternative strategy to get everybody tested. They explained that some pregnant women after counseling still refuse to go for HIV testing so they compulsorily do the test for them.

*“Some of the presenting pregnant women don’t want to come and test and they will manage to pass through to about 36 weeks of their gestation without testing. We spot some of them when they are about to deliver. We test them, admittedly, sometimes without their knowledge”* (Midwife, Key informant, health facility in Kumasi).

*“Those who are adamant or so difficult are those who listen to other people and they discourage them from testing, so those are the people we try our possible best to encourage and if they insist they won’t do it, then we either use force or do it without their knowledge* (Key informant interviews, , health facility in Kumasi).

### Health facility related factors influencing HTC

Lack of privacy times, inadequate information, ill treatment from staff and long waiting emerged as the facility related barriers to HTC among pregnant women.

### Long waiting time

Most of the women disclosed they waited too long at the health facility before being attended to so they left without taking the test. Other respondents explained there were queues for HTC and that discouraged them from taking the test and kept postponing.

A participant reiterated:

*“When you come here you wait too long. Before I finish seeing the midwife it is too late and I am hungry so I postpone but the situation is always the same” (pregnant woman, FGD, health facility in Kumasi*).

*“The problem here is the waiting time. Any day I come here I wait too long before being attended. So I am waiting till the day I will finish ANC early …. then I will go for the HIV test”* (*pregnant woman, FGD, health facility in Kumasi*).

### Relationship with health workers

Relationship with health workers was one of most cited barrier to HTC. Some of the participants disclosed they were not well treated by the nurses at the facility and that has discouraged them from participating in HTC.

A respondent emphasized this as follows:

*“As for this place they don’t treat us well at all. The nurses, especially the younger ones are so rude and they will be shouting at us for any small mistake we make. This is why I have not tested, because I always want to go home and leave this place”* (*pregnant woman, FGD, health facility in Kumasi*).

The participants explained when they present at health facility; they are in a hurry to see the midwife and go back home because the environment is not friendly and at times have to skip HTC because that will keep them waiting for long.

### Privacy and confidentiality

Some participants from one of the health facilities complained the testing is done in an open with no privacy. They explained the way and manner the test is done, and the results disclosed is what is deterring them from taking the test. A pregnant woman from that facility disclosed there is the likelihood of another patient getting to know your status due to the way and manner the process is handled.

This was expressed by one respondent as:

*“I decided not to take the test because it is too open to people. When we come the midwife calls the names and they sit around a table in the open where we are all waiting. When she finishes, she mentions some of the names and ask the rest to see her in the office and one can easily suspect something”* (*pregnant woman, FGD, health facility in Kumasi*).

The issue of privacy at HTC centre was buttressed by a health worker during the key informant interviews. She disclosed that some pregnant women refuse to take the test because they feel someone may get to hear or know their status.

“*Some refused the test because they say the room has not adequate privacy. They believe if they are positive, people will notice and they will be stigmatized”* (midwife, key informant interview, health facility in Kumasi).

*“The place we are working is not very good. Some patients upon seeing people around the counseling room refuse to enter the room. ….. People even sometimes hear us telling patients they are positive, and that deter others from coming”* (HIV counselor, key informant interview health facility in Kumasi).

### Inadequate information

Lack of information (not being told at ANC) emerged the greatest barrier to HTC as indicated by the participants. Some participants disclosed they had never heard of HIV testing and counseling since they started attending ANC and that was the reason why they had not taken the test. One of the participants reported:

*“Nobody has told me anything like that since I started coming here. I don’t know anything about HTC”* (Pregnant woman, FDG, health facility in Kumasi).

*“Please, have not been told to do it and I don’t know anything about it (HTC). That is why I have not done it”* (Pregnant woman, FDG, health facility in Kumasi).

The appropriateness of the information given hindered some of the pregnant women from undergoing HTC. One woman explained that she has been told by a midwife to go for HTC but she did not fully understand why she must go for it and when to do it.

*“Well I have been told but I wasn’t told when to do it (HTC). I didn’t know I have to do it now, I thought I have to wait till maybe when my pregnancy has progressed to the later stage, so I am just waiting till that time, ….. maybe the eighth month”* (Group 2).

In most of the facilities visited, HIV education and counseling was done for pregnant women once during ANC days and was done early in the morning. Those who come in later were most likely to miss the counseling session and therefore will not understand the need to go for HTC. A health worker from one facility disclosed;

*“I normally do the counseling for them once; early in the morning when I come to the facility, then I go and sit down and wait for those who will come so I do the testing for them”* (HIV counselor, key informant, health facility in Kumasi).

### Challenges faced by health workers

Health workers during in-depth interviews disclosed challenges they face in HIV testing and counseling. These challenges may influence the quality of services delivered and client’s perception. Some of the challenges cited include inadequate working space, shortage of working materials, lack of motivation and inappropriate siting of the counseling unit.

A health worker disclosed:

*“We are normally short of OraQuick, which we use as a confirmatory test. At times we ask patients to go and come because we have to send their blood samples to the reference laboratory at Komfo Anokye Teaching Hospital and many go and do not come back”* (HIV counselor, key informant interview, health facility in Kumasi).

*“The place we are working is not good enough. It is too small and we can’t even accommodate two people here. We normally ask them to wait outside which they are not comfortable of. Some of them will leave when you ask them to sit outside” (health staff, key informant interview, health facility in Kumasi*).

### Influence of perceptions of quality of care and HTC

The perception of pregnant women of the quality of care at the health facility significantly influenced their decision to undertake HIV counseling and testing. Pregnant women who did not feel attended to by health professionals had an odds of HTC that was 52% lower than those who fell attended to (OR = 0.48; p < 0.05), Table 2. Pregnant women who felt they were not listened to by health staffs were less likely to undertake HTC (OR = 0.52; p < 0.01). Similarly, as shown in Table 2, pregnant women who had negative perceptions of relationship with health staff, mistrust health workers on privacy, and confidentiality of counseling rooms were less likely to undergo HTC. Waiting for long time at the health facility also had significantly negative influence on HTC among pregnant women (OR = 0.43; p < 0.001). This negatively significant relationship was also observed in the multivariate analysis (AOR = 0.5; p < 0.005).

**Table 2:** Influence of perceptions of quality of care and HTC

**Variables**	**OR**	**AOR**
**I feel attended to at the facility**		
— Yes	1	1
— No	0.48 (0.30, 0.77)*	0.65 (0.33, 1.28)
**Listened to**		
— Yes	1	1
— No	0.52 (0.30, 0.87)**	1.36 (0.61, 3.05)
**You are treated with respect**		
— Yes	1	1
— No	0.56 (0.36, 0.85)**	1.0 (0.56, 1.78)
**You can trust the health workers**		
— Yes	1	
— No	1.08 (0.93, 1.26)	-
**Privacy and confidentiality**		
— Yes	1	1
— No	0.43 (0.25, 0.73)**	1.0 (0.52, 1.97)
**Do not wait long**		
— Yes	1	1
— No	0.49 (0.36, 0.66)***	0.50 (0.35, 0.72)***

*p < 0.05; **p < 0.01; ***p < 0.001 Main outcome = Utilization of HTC.

## Discussion

Health service utilization had been known to be greatly influenced by clients’ perception about factors that influence the provision of the service; clients with positive perceptions tend to utilize health services and vice versa [12,16,17]. In this study, we sought to gain an insight into how some health facility related factors influence utilization of HTC among pregnant women.

### Utilization of HTC

This study reported high HTC rate and this was consistent with studies by [18] in 2003 and 2005 where the acceptance rate was 97.0% and 73.0% respectively. It is also consistent with [19] in the Komfo Anokye Teaching Hospital (73.9%); [20] where HTC acceptance was reported to be 96.1% in Lagos and a study by [21] in Brazil. This was however inconsistent with other studies on HTC across the African continent which includes the study by [22] to assess voluntary counseling and testing among postpartum women in Botswana, 54%. This might however be as a result of the increased education over the few years on the need for pregnant women to undergo HIV testing and counseling to prevent infecting their babies.

### Health facility related factors influencing HTC

#### Inadequate information

Effective communication and counseling are important to improve awareness and make informed decision about available services. However, in the advent of weak health systems and limited resources, health providers may have had insufficient training, and their workloads may be so heavy that they do not find the time or space for counseling [23]. In this study, “Not being told” emerged the most cited reason for not testing for HIV (29.5%), supporting the fact that not all pregnant women who visit the facility benefit from HIV counseling service. Some participants in the qualitative study also disclosed they had never been given information at the facility regarding HTC and others said it was poorly explained to them. This could be explained by the non-routine nature of the HIV counseling at the health facilities and shows that some pregnant women could not make a decision to undergo HTC because they lacked information. This was evident in other study which found time for counseling to be insufficient [24] and the information often not adequate, and the quality of counseling lower for clients from the less-privileged segments of society [25].

This study also revealed that some clients were compelled to do the test. About 9% of the women who had tested indicated they took the test because they were told it was compulsory. Some participants in the qualitative study disclosed how the test was done without prior knowledge. Some of the health workers also disclosed they compelled the clients who were “stubborn” and still refused testing upon several counseling attempts. However, this could mean that the counseling was not adequate enough to translate into positive action. The act of compelling clients to undergo HTC however contradict the National Guidelines for PMTCT of HIV by the Ministry of Health Ghana [26] which states that the minimum amount of information to be given to ANC clients before HTC include “the right to refuse”. The guidelines further indicate specifically that the decision of a person to continually refuse the test should be respected and documented and their refusal should not compromise the quality of care they receive. This however could have adverse implications on the service and is an indication of the inadequacy of counseling and education given at the ANC. Interestingly, this practice is not limited to this setting. Studies of testing in Europe indicate that 10% to 20% of respondents are tested without their knowledge [27,28].

Another study in Brazil also indicates that, 29% of the women not counseled were not informed the test had been performed and 6.6% received the information that HIV testing was mandatory. About 6.2% among those counseled also received information that the testing was mandatory [8]. These situations could be more predominant in this setting where clients think they will receive improved care if they refuse to do what they have been ordered to by health staff or refusing could have adverse consequences [29,30].

#### Privacy and confidentiality

Issues of privacy protection and implementation of informed consent regarding HTC are of much concern to stakeholders. Efforts aimed at expanding HTC services have heightened awareness of the ethical dimension of HTC in health facilities. Lack of privacy at counseling centres has militated against effort to improve utilization of HTC. Some previous studies indicate that confidentiality is often compromised by established practices in health services [31] with poor women at a distinct disadvantage [32]. Previous studies in India, Thailand, the Philippines, and Indonesia, also reported breaches of confidentiality by health care workers (34% of respondents) [33]. Clients’ perceptions of level of privacy and confidentiality influence their willingness undergo HTC [34].

In this study, pregnant women who had negative perception about privacy and confidentiality at the facility were less likely to undergo HTC (OR = 0.43; p < 0.001). This was consistent with qualitative findings from the study by [35] which found lack of confidentiality for test result as a prevailing service provision related factor for refusal of HIV testing. In their study, respondents who were not sure about the maintenance of privacy at the time of counseling were five times more likely to refuse HIV testing as compared to those who responded that privacy was maintained at the time of counseling (OR = 5.2, 95% CI = 2.70, 9.79). This shows that privacy and confidentiality are pivotal in the improvement of HTC at the health facility level. This is much critical in a setting where stigmatization still remains predominant. HIV positives are very much stigmatized and people do not want others to know of their status. In the study by [36], fear of being seen and labeled as HIV victims was a reason behind some men’s refusal to undergo voluntary counseling and testing. Most of the men in the study expressed the feeling that they would go elsewhere with majority preferring VCT outside Bukonzo West where the counselors do not know them.

#### Fear of being at risk

Individuals’ reluctance to acknowledge that they are at risk has been documented in studies in the United States and Canada [37,38], the United Kingdom [39]; Brazil [40]; rural Tanzania [41] and Ethiopia [42]. Predominance of stigma in societies especially in this setting has also resulted in fear for HTC in most people, especially women. In this study, some respondents (10.7%) cited fear of being positive as their reason for not undergoing HTC. Other (8.7%) also were afraid of being stigmatized if they test positive. Most women are afraid of social consequences of the illness—rejection by loved ones, loss of job or housing, discrimination, and violence [43]. Others are also afraid they will be blamed by their partners or divorced if they become the first to disclose a positive status to their partners. Consistently, other studies by [22] and [44] reported fear of testing positive as the greatest barrier to HIV testing. This resulted in a gap between intentions and actual number of respondents who are able to undergo HTC. In a study of mothers in Zaire for instance, as high as 70% expressed willingness to undergo HTC, but only 2% followed up 12 months later [45].

#### Long waiting time

Long waiting time was also a major health facility related barrier to HTC in the Kumasi metropolis. Most participants from all selected health facilities indicated their dissatisfaction with the waiting time. Participant’s perception about waiting time had a significantly relationship with undergoing HIV testing and counseling in both the bivariate and multivariate analysis. Participants in the FGDs also explained they waited too long at ANC and therefore could not have time to go for HTC. Some pregnant women explained they get stressed up and hungry when they wait for long and therefore are in hurry to go home after they are attended to by the midwife. The result is also consistent with a study by [46] where long waiting periods for results resulting from overburdened health staff were also found to be barriers to HTC. The study by [12] also reported long waiting time as a major barrier to accessing ART for PMTCT among HIV positive women.

#### Relationship with health workers

Issues surrounding counseling represent a major challenge for the utilization of HTC. Health workers have their own peculiar challenges, which could influence their relationship and communication with clients and encourage them to adopt appropriate behaviours. They may be reluctant to be tested as a result of stigma [47], fear contamination, pessimistic and doubtful of their ability to provide care [48,49]. Conversely, a good rapport between providers and clients is an important determinant of patients’ acceptance of clinic-based interventions, including testing [50].

In this study, respondents who believed they were not well attended to and were not treated with respect by the health workers were less likely to undergo HTC. Participants in the qualitative study also narrated ill treatment by health workers which discouraged them from undergoing HTC. Supporting providers to gain clients’ trust is therefore an important step in improving uptake of HTC among pregnant women. Health workers in this study reported inadequate working space, shortage of working materials, lack of motivation and inappropriate citing of the counseling unit as some challenges encountered in providing PMTCT services at the ANC. Efforts to improve uptake of HTC should therefore focus attention on providers to define the needed services and ascertain the training, time, and resources necessary to deliver them.

### Limitations

This study might not have studied all factors that could militate against HTC at the health facility. The authors however assumed that the factors considered are important issues especially in this setting and going further to assess the views of health workers provided a better understanding of situations at the facility. Again, some respondents might not have been open with information regarding the facility and health workers and that could result in information bias. Interviews and discussions were however conducted in private rooms and in closets and participants were assured of the privacy with information they provided. Finally, social desirability may have influence some of the responses from study participants. However, the study triangulated the responses by interviewing both clients and providers using different interviewing techniques consistent with other studies within the scope of this study. Hence conclusions are both valid and reliable.

## Conclusions

Linking HTC to ANC has not yielded many benefits as most presenting pregnant women at ANC do not utilize ANC due to health facility related factors such as poor health education and information, lack of privacy and confidentiality, long waiting time or poor health staff turnaround time and poor health staff-client relationship at ANC. Ensuring closer supervision of health workers and provision of the needed work inputs and training of ethics of HTC could improve utilization of HTC linked to ANC.

## Competing interests

The authors declared no conflict of interests.

## Authors’ contributions

The study was conceived and designed by all authors. DB and GDK jointly collected the data under supervision by PA-B and EAA. DB led the data analysis and interpretation of the study findings. All authors reviewed and critically revised the manuscript for important intellectual content and agreed to submit the manuscript for publication. All authors read and approved the final manuscript.

## Pre-publication history

The pre-publication history for this paper can be accessed here:

http://www.biomedcentral.com/1472-6963/14/267/prepub
